# Haemodynamic trajectories around prehospital adrenaline infusion start after return of spontaneous circulation

**DOI:** 10.1016/j.resplu.2026.101367

**Published:** 2026-05-21

**Authors:** Pieter Francsois Fouche, Emily Nehme, Sam Burton, Benjamin Meadley, David Anderson, Belinda Flanagan, Dion Stub, Ziad Nehme

**Affiliations:** a University of Tasmania, School of Paramedicine and Public Safety, Hobart, Australia; b Ambulance Victoria, Melbourne, Australia; c School of Public Health and Preventive Medicine, Monash University, Melbourne, Australia; d Department of Paramedicine, Monash University, Melbourne, Australia

**Keywords:** Cardiac arrest, Return of spontaneous circulation, Blood pressure, Prehospital, Out-of-hospital

## Abstract

**Background:**

Post-return of spontaneous circulation (ROSC) hypotension is common and associated with worse outcomes, but the immediate haemodynamic response to prehospital adrenaline infusion is poorly described. We examined minute-level blood pressure trajectories around documented infusion initiation in out-of-hospital cardiac arrest (OHCA) patients who achieved sustained ROSC and arrived at hospital with a pulse present.

**Methods:**

We performed a retrospective linked cohort study of adult emergency medical services-treated out-of-hospital cardiac arrest cases in Victoria, Australia, from 2019 to 2023, who achieved sustained ROSC, had available linked ZOLL® monitor-defibrillator data, and arrived at hospital with a pulse present. Patients started on prehospital adrenaline infusion within 60 min of ROSC were included. Minute-level mean arterial pressure (MAP) and systolic blood pressure (SBP) trajectories from 10 min before to 20 min after infusion start were analysed using segmented mixed-effects models adjusted for pre-infusion mean pressure and pre-infusion slope.

**Results:**

Of 3655 eligible adult ROSC cases with linked monitor data and pulse present on hospital arrival, 1920 received prehospital adrenaline infusion within 60 min of ROSC. Blood pressure generally declined in the 10 min before infusion, reached a nadir at or near infusion start, and then rose over the next 20 min. In the primary models, MAP fell by 1.64 mmHg/min before infusion and rose by 1.15 mmHg/min after infusion, a slope change of 2.79 mmHg/min (95% CI 2.48–3.09). SBP fell by 2.25 mmHg/min before infusion and rose by 1.71 mmHg/min after infusion, a slope change of 3.96 mmHg/min (95% CI 3.55–4.38). These changes reflected a marked reversal in trajectory around infusion start. Descriptively, mean MAP increased from 81.2 mmHg at 5 min after infusion start to 95.4 mmHg at 20 min, while mean SBP increased from 107.9 to 128.4 mmHg.

**Conclusions:**

Documented adrenaline infusion start coincided with reversal of a falling blood pressure trajectory and a sustained rise in MAP and SBP over the next 20 min. These findings help define prehospital post-ROSC adrenaline arterial-pressure responsiveness and support further study of vasopressor timing, targets, and trial design.

## Introduction

Post-cardiac arrest syndrome commonly includes an early phase of myocardial dysfunction, vasoplegia and haemodynamic instability after return of spontaneous circulation (ROSC).^1^, ^2^ Avoiding hypotension is therefore a central goal of post-resuscitation care, and contemporary guidelines recommend maintaining at least a minimum mean arterial pressure (MAP) around 65 mmHg while acknowledging uncertainty about the optimal blood pressure target and the best vasopressor strategy to achieve it.^3^, ^4^ Vasopressor support is frequently required, but the potential haemodynamic benefits of catecholamines must be balanced against myocardial oxygen demand and arrhythmia, and evidence to guide the timing of specific infusions after cardiac arrest remains limited.^2^, ^3^, ^4^

Existing post-ROSC blood pressure studies answer related but distinct questions. ICU blood-pressure target trials address protocolised haemodynamic management after hospital admission, whereas the immediate prehospital phase concerns unstable minutes after ROSC, before invasive monitoring and ICU-based optimisation are established.^3^, ^4^, ^5^ Yet observational studies continue to show that circulatory shock, lower blood pressure and greater cumulative hypotension exposure are associated with worse outcomes after ROSC.^6^, ^7^, ^8^ This leaves a narrower descriptive gap: the timing, direction, and variability of arterial-pressure change around prehospital vasopressor commencement.

That phase remains poorly described in most studies. Much of the literature on post-ROSC blood pressure has relied on a single value at hospital handover or hospital admission.^6^, ^9^ Such measurements compress a dynamic treatment period and cannot distinguish untreated early shock from physiology already modified by fluids or vasopressors. By contrast, recent minute-level monitor-defibrillator data show that even brief early hypotension and longer time below conventional systolic blood pressure and MAP thresholds are associated with lower survival after ROSC, supporting the idea of an early vulnerability window in the prehospital setting.^7^

The difficulty is that routine observational data do not cleanly answer whether a prehospital adrenaline infusion should be started at all. Infusion initiation is closely tied to evolving blood pressure, recent trajectory, co-interventions and clinician judgement. As a result, simple comparisons of infusion versus no infusion are highly vulnerable to confounding by indication and time-dependent bias, including immortal-time and related resuscitation-time problems.^10^ In linked registry, electronic clinical record and monitor data, important markers of illness severity remain incompletely measured, while later outcomes such as survival or neurological recovery are shaped by many post-exposure processes well beyond the infusion decision.

Given these limitations, a narrower physiological question is more useful and defensible than a broader causal treatment comparison: what arterial-pressure trajectory is observed around the start of a prehospital adrenaline infusion in patients who receive it? This remains clinically relevant because arterial pressure is an intermediate physiological target on the pathway between post-ROSC shock and patient outcomes. Among patients who received a prehospital adrenaline infusion after sustained ROSC, this study examined changes in mean arterial pressure and systolic blood pressure around infusion initiation

## Methods

This study is reported in accordance with the STROBE guidelines for cohort studies.^11^

### Study design and setting

This retrospective observational study used linked registry, clinical record, medication, and monitor-defibrillator data from Ambulance Victoria, the statewide emergency medical service for Victoria, Australia. Ambulance Victoria operates a two-tiered system staffed by Advanced Life Support paramedics and Mobile Intensive Care Ambulance paramedics. Post-ROSC care is clinician-directed under statewide protocols and prioritises rapid transport while stabilising oxygenation, ventilation, and perfusion.^12^ Hypotension after ROSC is initially managed with judicious intravenous fluid administration; total fluid during cardiac arrest and post-ROSC care generally does not exceed 20 mL/kg unless correcting suspected hypovolaemia, and smaller aliquots may be used in patients at risk of fluid overload. Intravenous fluid therapy was not restricted by this study during or after infusion commencement; continuation, additional aliquots, or cessation were clinician-directed and captured only when documented in the clinical record. If hypotension persists despite initial fluid, or if profound hypotension is imminent, vasopressor support may be escalated according to clinician judgement and contemporaneous protocol, including vasopressor boluses and adrenaline infusion via syringe pump. The exact decision to commence and titrate infusion was not assigned by the study protocol. Accordingly, the present study was designed as a within-patient pharmacodynamic analysis centred on infusion start.

### Data sources and participants

We linked the Victorian Ambulance Cardiac Arrest Registry, electronic patient care records, structured medication records, and ZOLL X Series® monitor-defibrillator files for OHCA cases during the 11 February 2019–14 December 2023 study period. The source cohort comprised adult EMS-treated OHCA patients with ROSC, retrievable ZOLL® data, and pulse present on hospital arrival. Patients aged under 18 years and traumatic arrests were excluded.

For the present analysis, we further restricted the cohort to patients with a documented prehospital adrenaline infusion started within 60 min of ROSC. Pulse present on hospital arrival was used as an operational end-of-prehospital-care cohort criterion rather than a formal duration-based definition of sustained ROSC. Infusion start was defined using the first non-zero documented adrenaline infusion-rate timestamp where available, otherwise the first recorded infusion-start timestamp. Time to infusion was calculated from the recorded ROSC time. The study did not standardise adrenaline infusion rates. Rates were chosen and documented during routine care, and we did not have a verified minute-by-minute pump record showing every rate change. The medication records provided patient-level summaries, including the median, mean, maximum, and number of documented rate entries. Therefore, the main exposure was the documented infusion-start minute, while infusion-rate data were used only as descriptive context.

### Minute-level physiology and time-series construction

Time-stamped monitor streams for systolic, diastolic, and mean arterial pressure were extracted from ZOLL® files. Blood pressure measurements were primarily automated non-invasive oscillometric cuff readings recorded by the ZOLL X Series® monitor-defibrillator. We did not have monitor-specific biomedical calibration records, cuff-size records, or paired invasive arterial pressure validation data, so measurement accuracy was assumed to reflect routine operational device performance rather than independently verified study calibration. Raw measurements were aligned to ROSC and converted to integer whole minutes after ROSC, with minute 0 representing 0–<1 min after ROSC. Implausible values were removed using prespecified bounds: non-positive SBP, DBP, and MAP values; SpO_2_ <50% or >100%; ETCO_2_ equal to 0; and respiratory rate <2 or >100 breaths/min. No carry-forward or carry-backward imputation was used.

Because non-invasive cuff measurements can be repeated by the monitor without a new inflation cycle, primary minute-level SBP and MAP values were defined as the mean of unique SBP and MAP values observed within each patient-minute. A repeated-hold suppression rule was then applied across adjacent minutes to truncate prolonged runs of identical SBP or MAP values likely to reflect monitor carry-over. These rules were intended to reduce the chance that repeated stored cuff values were counted as new blood pressure measurements. However, the data remain intermittent non-invasive cuff readings, not continuous beat-to-beat arterial pressure. Monitor processing and minute-level binning broadly followed our prior ROSC-aligned pipeline, modified here so that the analytic anchor was infusion start rather than ROSC.^7^ Structured medication records were used to reconstruct minute-level post-ROSC bolus adrenaline exposure. Additional time-varying covariates were created from procedure and medication timestamps, including recent normal saline administration, recent intubation, and recent sedation or paralysis.

### Around infusion-start windows and derived measures

The primary modelling window spanned 10 min before to 20 min after infusion start, with minute 0 defined as the minute of infusion start. The pre-infusion model window was restricted to the 10 min immediately before infusion start to capture the haemodynamic trajectory most proximate to the clinical decision to commence infusion, while limiting contamination from earlier post-ROSC stabilisation and preserving usable data in patients with earlier infusion initiation. Patient-level pre-infusion mean pressure was calculated over minutes −10 to −1, and patient-specific pre-infusion and post-infusion slopes were estimated by simple linear regression of pressure on relative time within the pre and post windows, respectively. To support slope estimation, patients were required to contribute at least two observed BP recordings in the pre-infusion window and at least two observed BP recordings in the post-infusion window for the relevant MAP or SBP model. This was only a minimum data requirement for estimating patient-level slopes. It did not mean that only four blood pressure values were used per patient. After cleaning, all available patient-minutes within the analysis window contributed to the mixed-effects trajectory models.

Descriptive change summaries were also derived at approximately +5, +10, +15, and +20 min by averaging observed BP values within a ±2-min band around each target minute. Secondary exact-time summaries were calculated from raw irregular BP observations using trapezoidal integration, including time-weighted average pressure and pressure-deficit burden below prespecified thresholds.^13^ Prespecified hypotension thresholds were MAP <65 mmHg and SBP <90 mmHg.

The principal pharmacodynamic parameter of interest was change in BP trajectory after infusion start. For descriptive summaries, we also prespecified two binary rise definitions: an increase in MAP of at least 5 mmHg at approximately 10 min after infusion start, and an increase in SBP of at least 10 mmHg at approximately 15 min after infusion start. We also summarised whether the patient-specific MAP slope changed in a positive direction. These rise summaries were descriptive and were not intended to classify patients as true pharmacological responders or non-responders.

### Statistical analysis

Continuous variables are summarised as mean with standard deviation or median with interquartile range, as appropriate, and categorical variables as count with percentage. The main analyses used segmented mixed-effects linear models with patient-specific random intercepts to model minute-level MAP and SBP around infusion start. Fixed effects included relative time, an indicator for the post-start period, and a post-start time term, allowing estimation of the pre-infusion slope, any immediate level change at infusion start, the post-infusion slope, and the change in slope after infusion initiation. The main pharmacodynamic parameter of interest was the post-start slope change, because a continuously titrated infusion is more plausibly expected to alter trajectory over the next several minutes than to produce an instantaneous step change.

The primary model adjusted for each patient’s pre-infusion mean pressure and pre-infusion slope, so that around-start response was interpreted conditional on the level and direction of haemodynamic change immediately before infusion. Because post-start co-interventions may lie on or near the pathway through which an infusion acts, the primary model did not adjust for care delivered after infusion start. A prespecified sensitivity model added one-minute-lagged time-varying indicators for recent bolus adrenaline, recent normal saline, recent intubation, and recent sedation or paralysis. This lagged specification avoided use of future information at a given minute, but it was treated as a sensitivity analysis rather than as the primary estimate because these variables may be affected by the same clinical deterioration that prompted infusion.

As a further sensitivity analysis, we refit the primary MAP and SBP segmented mixed models with an autoregressive residual correlation structure of order 1, specified on the observed minute spacing within each patient, to account for serial dependence between adjacent blood pressure measurements. This analysis assessed whether the main around-start slope-change estimates were materially altered by within-patient residual autocorrelation.

Prespecified falsification and robustness analyses examined early and late pre-infusion slopes separately, inserted a placebo breakpoint at 5 min before actual infusion start, repeated the segmented models using alternative windows of −5 to +15, −10 to +15, and −10 to +30 min, and repeated the segmented models after shifting the breakpoint 3 and 5 min earlier than the documented infusion-start minute in fake-start analyses. These checks were used to assess whether any apparent post-start turn in trajectory could be explained by pre-existing rebound or arbitrary breakpoint placement. Each model used complete cases for the variables required by that analysis, so denominators varied across MAP and SBP models and across derived summaries.

Additional exploratory analyses examined whether the blood pressure response differed by pre-infusion haemodynamic status, recent treatments, time from ROSC to infusion start, and documented infusion-rate category. Implausible blood-pressure values were also summarised. These analyses were interpreted descriptively because the subgroups, treatments, timing, and documented rates reflected clinical care rather than randomised treatment assignment.

Analyses were performed in Stata® version 19 (StataCorp, College Station, TX, USA). Estimates are reported with 95% confidence intervals.

### Ethics

Ethics approval was obtained from the Monash University Human Research Ethics Committee (ID 41476). Ambulance Victoria Research Committee approved the study (R24-001).

## Results

### Cohort

**Fig. 1:**
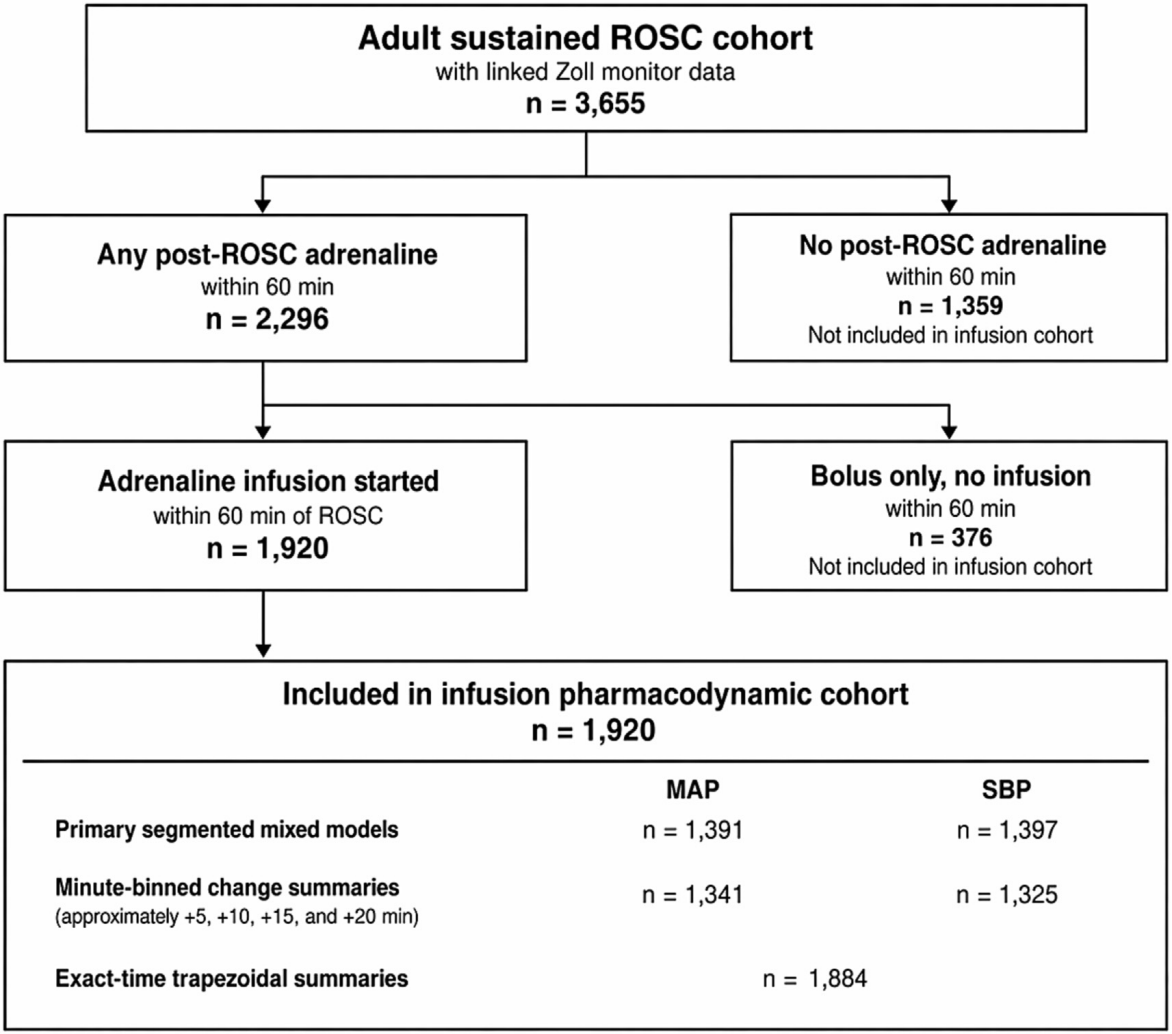
**Patient selection and analytic cohorts**. Adult OHCA cases with ROSC, linked ZOLL monitor data, and pulse present on hospital arrival formed the source cohort. The main analytic cohort comprised patients with documented prehospital adrenaline infusion initiation within 60 min of ROSC. Exact-time paired change summaries required usable blood pressure observations on both sides of infusion start.

Of 3655 adults with ROSC, linked ZOLL® monitor data, and pulse present on hospital arrival, 1920 had a prehospital adrenaline infusion started within 60 min of ROSC and formed the infusion-starter cohort (Fig. 1). Median age was 65 years, 66.3% were male, median downtime (low-flow plus no-flow) was 30 min, and VF/VT was the initial rhythm in 41.0%. Infusion was started a median of 11 min after ROSC. Before infusion start, 67.9% received at least one post-ROSC adrenaline bolus. By the time the infusion commenced, the cumulative documented bolus dose was a median of 60 µg (IQR 0–400). The median documented infusion rate was 20 µg/min (IQR 10–50). Most patients had only one documented numeric infusion-rate entry (median 1, IQR 1–3), so infusion-rate analyses were descriptive and were not treated as minute-by-minute dose–response analyses. Crystalloid had been administered in 50.5% of cases, and 79.1% of patients had been intubated. Primary segmented models included 1391 patients for MAP and 1397 for SBP. Exact-time trapezoidal summaries were available for 1884 patients, and paired exact-time pre-post summaries included 1381 MAP and 1387 SBP observations. Further cohort characteristics and treatment context are summarised in Table 1 and Supplementary Table S1. Non-positive BP values were negligible in the linked ZOLL® extract: 0 SBP values, 0 MAP values, and 10 DBP values among 553,177 records, 0.002%.

**Table 1:** Characteristics of the infusion-starter cohort and treatment context at infusion start.

**Variable**	**Infusion-starter cohort** **(*N* = 1920)**
**Patient and arrest characteristics**	
Age, years	65 (53–76)
Male sex	1273 (66.3)
Downtime, min	30 (23–40)
Public location	359 (18.7)

**Witness status**	
EMS witnessed	289 (15.1)
Not witnessed	466 (24.3)
Public witness	1159 (60.6)
Bystander CPR	1294 (67.4)

**Initial rhythm**	
VF/VT	785 (41.0)
PEA	539 (28.1)
Asystole	519 (27.1)
Non-shockable/other	72 (3.8)
Presumed cardiac cause	1431 (74.5)
Total shocks	1 (0–3)

**Treatment context at infusion start**	
Time from ROSC to infusion start, min	11 (6–20)
Any prior adrenaline bolus prior to infusion start	1303 (67.9)
Cumulative bolus dose prior to infusion start, mg	0.06 (0.00–0.40)
Any crystalloid before infusion start	969 (50.5)
Intubated before infusion start	1519 (79.1)
Any rocuronium, midazolam, ketamine, or fentanyl bolus before infusion start	527 (27.4)

**Pre-infusion haemodynamics**	
Pre-window mean MAP, mmHg	84.0 (70.3–99.6)
Pre-window minimum MAP, mmHg	73.0 (59.0–89.0)
Pre-window mean SBP, mmHg	111.8 (92.0–133.7)
Pre-window minimum SBP, mmHg	95.0 (76.0–118.0)
Pre-infusion MAP slope, mmHg/min	−1.15 (−4.16–0.00)
Pre-infusion SBP slope, mmHg/min	−1.67 (−5.97–0.00)

Values are median (IQR) or n (%). Percentages are column percentages. “Start” refers to the documented infusion-start minute. Available denominators differed from the header N for downtime (1919), witness status (1914), initial rhythm (1915), pre-window MAP summaries (1505), pre-window SBP summaries (1512), pre-infusion MAP slope (1391), and pre-infusion SBP slope (1397).

### Haemodynamic trajectories

**Fig. 2:**
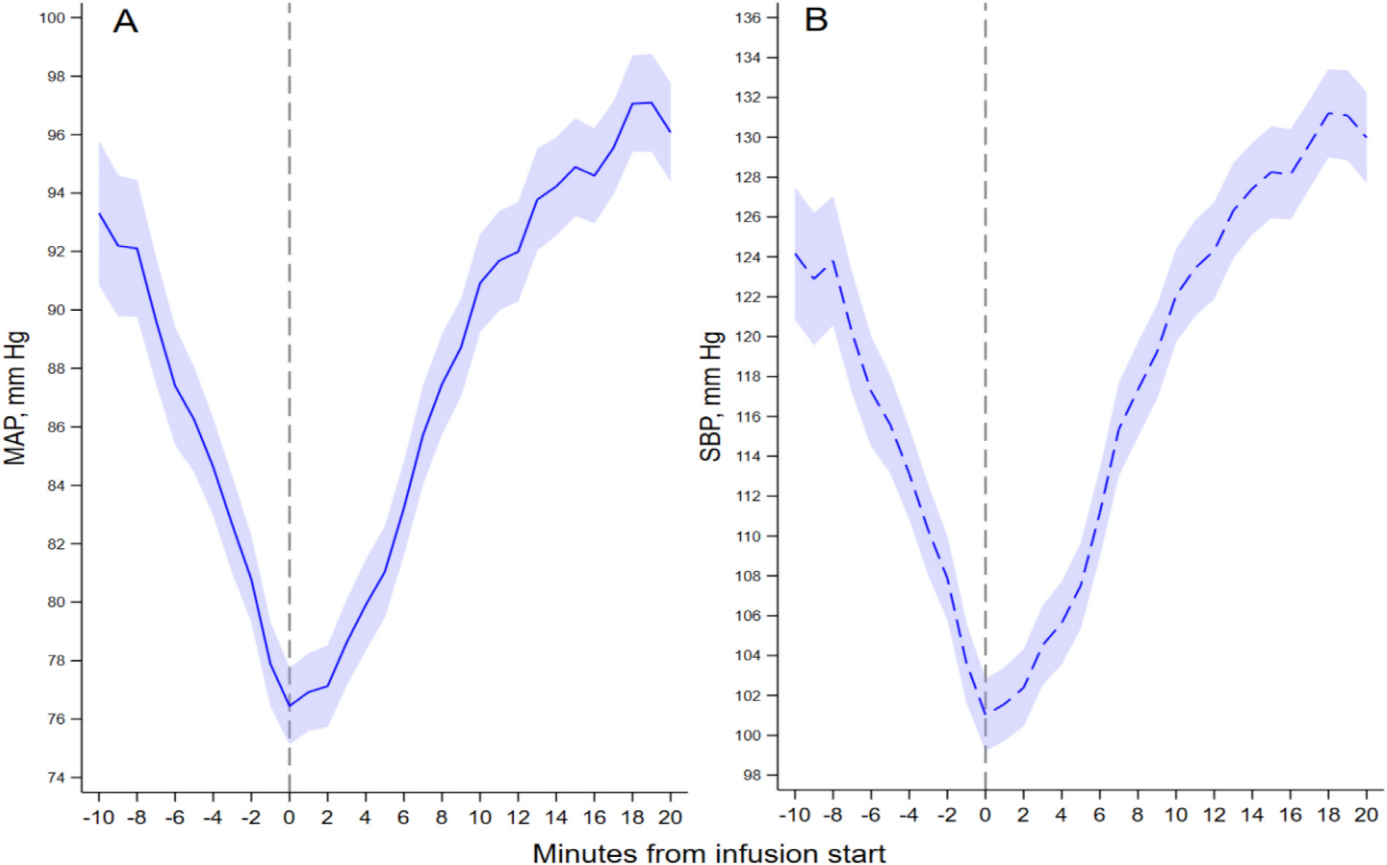
**Observed blood pressure trajectories around prehospital adrenaline infusion start**. (A) Mean arterial pressure. (B) Systolic blood pressure. Lines show observed minute-level means; shaded bands show 95% confidence intervals for the observed means. Minute 0 (dashed line) denotes infusion start. Values are based on cleaned intermittent non-invasive blood pressure observations and should not be interpreted as continuous beat-to-beat arterial pressure.

Before infusion, blood pressure was generally falling. Observed trajectories showed a consistent reversal around infusion initiation (Fig. 2): MAP and SBP declined during the 10 min before start, reached a nadir at or near minute 0, and then rose over the subsequent 20 min. In the primary segmented mixed model, infusion start was associated with a 2.79 mmHg/min change in MAP slope (95% CI 2.48–3.09; *p* < 0.001) and a 3.96 mmHg/min change in SBP slope (95% CI 3.55–4.38; *p* < 0.001) (Table 2). Pre-infusion slopes were negative (−1.64 mmHg/min for MAP and −2.25 mmHg/min for SBP), whereas post-infusion slopes were positive (1.15 and 1.71 mmHg/min, respectively). There was no clear immediate level change at the documented start minute: estimated changes were −0.68 mmHg for MAP (95% CI −2.20 to 0.83; *p* = 0.374) and −1.75 mmHg for SBP (95% CI −3.86 to 0.37; *p* = 0.106).

**Table 2:** Segmented mixed-model estimates for MAP and SBP trajectories around prehospital adrenaline infusion start.

**Outcome and model**	**Estimate (95% CI)**	***p*-value**
**MAP models**		
**Primary model**		
Slope change after infusion start	2.79 (2.48–3.09)	<0.001
Pre-infusion slope	−1.64 (−1.91 to −1.37)	<0.001
Post-infusion slope	1.15 (1.05–1.25)	<0.001
Immediate level change at start	−0.68 (−2.20 to 0.83)	0.374
**Sensitivity analysis**		
Slope change after infusion start	2.50 (2.19–2.81)	<0.001
Pre-infusion slope	−1.52 (−1.79 to −1.24)	<0.001
Post-infusion slope	0.99 (0.89–1.09)	<0.001
Immediate level change at start	−0.29 (−1.81 to 1.22)	0.705

**SBP models**		
**Primary model**		
Slope change after infusion start	3.96 (3.55–4.38)	<0.001
Pre-infusion slope	−2.25 (−2.63 to −1.88)	<0.001
Post-infusion slope	1.71 (1.58–1.85)	<0.001
Immediate level change at start	−1.75 (−3.86 to 0.37)	0.106
**Sensitivity analysis**		
Slope change after infusion start	3.57 (3.14–4.00)	<0.001
Pre-infusion slope	−2.09 (−2.46 to −1.71)	<0.001
Post-infusion slope	1.49 (1.35–1.63)	<0.001
Immediate level change at start	−1.19 (−3.32 to 0.94)	0.273

Coefficients are in mmHg or mmHg/min. Primary models adjusted for the patient-specific pre-infusion slope and pre-window mean pressure. Lagged sensitivity models additionally adjusted for 1-min lagged recent bolus adrenaline, crystalloid proxy, intubation, and sedative/paralytic exposure. MAP models used 25,874 patient-minutes from 1391 patients; SBP models used 26,078 patient-minutes from 1397 patients.

The primary MAP model used 25,874 patient-minutes from 1391 patients, and the primary SBP model used 26,078 patient-minutes from 1397 patients. Patients contributed a median of 5 observed blood pressure recordings in the 10-min pre-infusion window and 13 in the 20-min post-infusion window for both MAP and SBP. In the lagged sensitivity models, the estimated slope change remained 2.50 mmHg/min for MAP and 3.57 mmHg/min for SBP (Supplementary Table S4). Separately, descriptively, mean MAP increased from 81.2 mmHg at approximately 5 min after infusion start to 95.4 mmHg at 20 min, while mean SBP increased from 107.9 to 128.4 mmHg over the same interval. Results were also unchanged in AR(1) sensitivity analyses, with slope-change estimates of 2.83 mmHg/min for MAP and 4.09 mmHg/min for SBP, again without a clear immediate level change; residual autocorrelation was high for both outcomes (*rho* 0.87 and 0.88).

### Patient-level change summaries and heterogeneity of early pressure rise

Despite the overall reversal in blood pressure trajectory around infusion start, individual responses were highly variable. At approximately 10 min, MAP changed by a mean of 4.03 mmHg (SD 30.16). At approximately 15 min, SBP changed by a mean of 11.67 mmHg (SD 42.86) (Supplementary Table S2). Mean post-minus-pre slope changes were 2.93 mmHg/min for MAP and 4.33 mmHg/min for SBP.

Simpler within-patient pre-post summaries showed the same overall pattern, although the average changes were smaller. Mean change in time-weighted average pressure was 1.96 mmHg (SD 23.92) for MAP and 2.74 mmHg (SD 33.72) for SBP. Patients also had less overall exposure to hypotension after infusion start, reflected by reductions in time-standardised hypotension burden of −0.58 for MAP and −1.01 for SBP (Table 3).

**Table 3:** Time-weighted haemodynamic change summaries and prespecified rise-definition frequencies around infusion start.

**Metric**	***N***	**Mean (SD) or *n*/*N* (%)**	**Median (IQR)**
**Exact-time paired summaries**			
Change in time-weighted average MAP, mmHg	1381	1.96 (23.92)	0.57 (−12.60–17.30)
Change in time-weighted average SBP, mmHg	1387	2.74 (33.72)	1.82 (−18.39–24.56)
Change in time-standardised MAP deficit burden	1381	−0.58 (5.57)	0.00 (0.00–0.63)
Change in time-standardised SBP deficit burden	1387	−1.01 (8.56)	0.00 (−0.65–1.31)

**Prespecified rise definitions**			
MAP rise ≥5 mmHg at approximately +10 min	1341	627/1341 (46.8)	–
SBP rise ≥10 mmHg at approximately +15 min	1325	684/1325 (51.6)	–
Positive MAP slope change	1367	1018/1367 (74.5)	–

Exact-time paired summaries were based on trapezoidal integration of irregular blood pressure observations. Time-standardised deficit burden represents the average amount by which blood pressure was below the prespecified hypotension threshold across the analysis window, standardised for window length; thresholds were MAP <65 mmHg and SBP <90 mmHg. Prespecified rise definitions were MAP increase ≥5 mmHg at approximately +10 min, SBP increase ≥10 mmHg at approximately +15 min, and positive MAP slope change. Negative change values indicate less overall exposure to hypotension after infusion start.

Using these prespecified rise definitions, 46.8% of patients had a MAP increase of at least 5 mmHg at approximately 10 min after infusion start. The corresponding frequency was 51.6% for an SBP increase of at least 10 mmHg at approximately 15 min, and 74.5% for a positive MAP slope change, indicating an upward change in trajectory even when the absolute pressure rise was smaller (Table 3). These frequencies show that an early pressure rise was common but not universal. Patients meeting the MAP-rise definition started from lower pre-infusion pressures than those who did not (Supplementary Table S3). This difference was also evident in the exact-time paired summaries, where patients meeting the MAP-rise definition had positive mean time-weighted average changes in MAP and SBP, whereas those not meeting it had average declines.

**Fig. 3:**
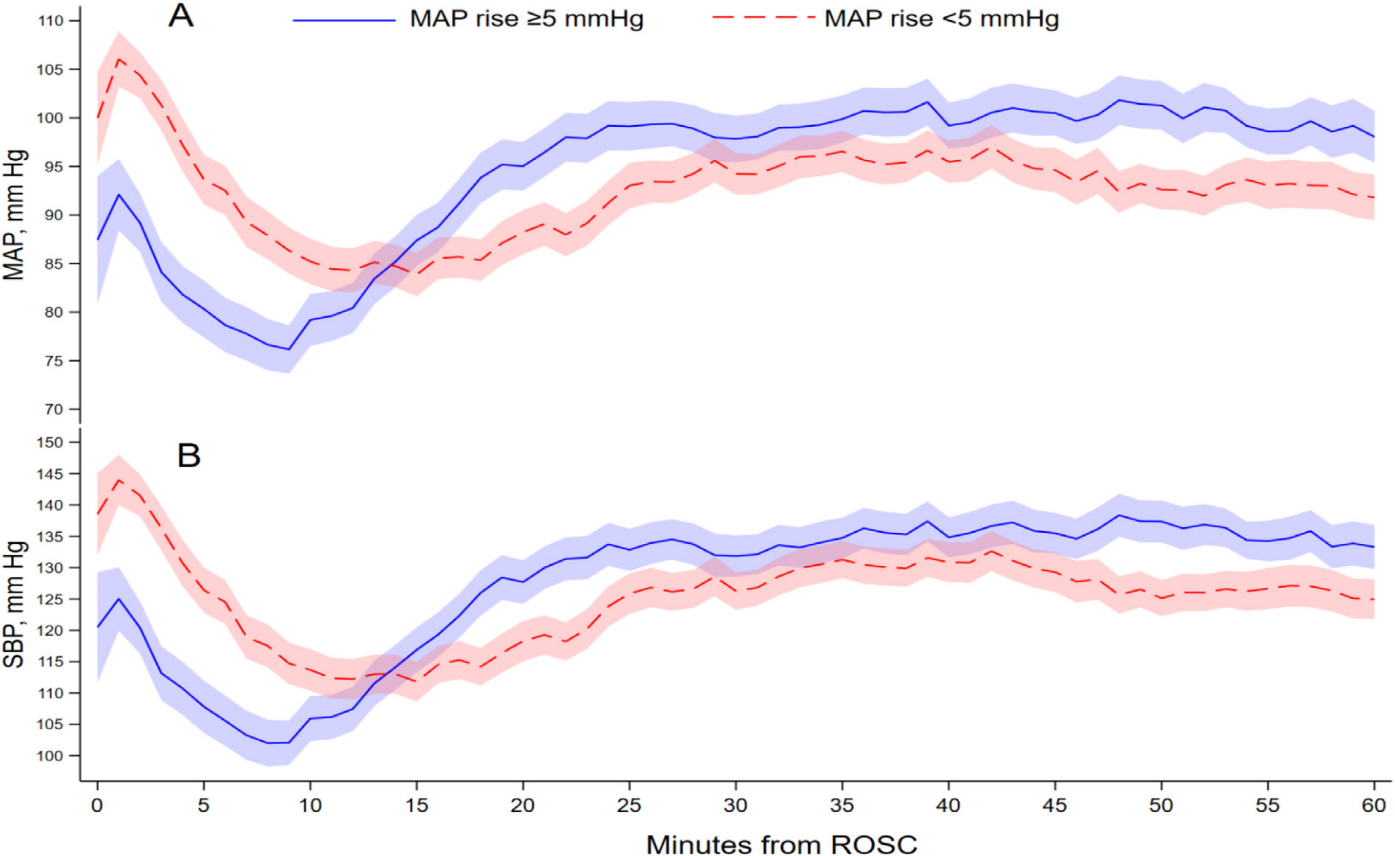
**ROSC-anchored blood pressure trajectories by MAP-rise category**. (A) Mean arterial pressure. (B) Systolic blood pressure. Patients were grouped according to whether MAP increased by at least 5 mmHg at approximately +10 min after infusion start. Curves show observed minute-level means with 95% confidence intervals. This post-start grouping is descriptive and should not be interpreted as a baseline predictor analysis.

The difference between positive slope change and absolute MAP rise shows that the rise definitions are descriptive, not true responder phenotypes. Exploratory subgroup summaries showed substantial heterogeneity in early MAP response (Supplementary Table S6). Patients with pre-infusion MAP <65 mmHg had the largest absolute rise: 85.6% had a MAP increase of at least 5 mmHg, with mean ΔMAP of 29.1 mmHg at approximately +10 min. In contrast, among patients with pre-infusion MAP ≥85 mmHg, only 25.6% had a MAP increase of at least 5 mmHg, although 76.3% still had a positive MAP slope change. These findings support heterogeneity in early arterial-pressure responsiveness and should be interpreted descriptively, not as causal subgroup effects (Fig. 3).

### Robustness analyses

Robustness analyses supported a broad around-start trajectory reversal, while also showing that the signal was not confined to one exact recorded minute. Pre-infusion slopes remained negative in both earlier and later pre-start periods, placebo-breakpoint models at −5 min did not identify a pseudo-turn, and positive slope-change estimates persisted across alternative windows and fake-start checks. Attenuation when the breakpoint was shifted earlier is compatible with a physiological turn occurring around the documented start period, with some imprecision in the recorded infusion-start time (Supplementary Table S5).

## Discussion

This within-patient pharmacodynamic analysis found that, in prehospital ROSC patients, MAP and SBP were generally falling before adrenaline infusion initiation, then increased after the infusion was started. In the primary segmented models, infusion start was associated with a MAP slope change of 2.79 mmHg/min and an SBP slope change of 3.96 mmHg/min. Pre-infusion slopes were negative, post-infusion slopes were positive, and there was no clear immediate level jump at minute zero.

The absence of a clear immediate level change, together with negative pre-infusion slopes and positive post-infusion slopes, suggests that the main signal was a reversal in blood pressure trajectory around infusion start rather than an abrupt step increase at minute zero. This pattern is more consistent with a progressive haemodynamic response over several minutes than with an immediate bolus-like effect, which is plausible for a low-rate infusion. Even so, this remains a temporal physiological signal around infusion initiation, not proof that the infusion itself caused the entire rise in blood pressure. A rise in blood pressure in this setting is clinically relevant, but it does not by itself establish benefit in survival or neurological outcome. Nor does a pressure rise show whether flow improved; this study did not measure cardiac output, systemic vascular resistance, or myocardial work.

This interpretation fits current understanding of post-resuscitation shock. After ROSC, haemodynamics are unstable because myocardial dysfunction, vasoplegia, relative hypovolaemia, and impaired autoregulation coexist and evolve over minutes to hours.^1^, ^2^ Current guidance therefore emphasises avoidance of hypotension, while acknowledging that the optimal target and timing of prehospital vasopressor support remain uncertain.^3^, ^4^ Observational studies have consistently linked lower post-ROSC blood pressure, circulatory shock, and greater hypotension exposure with worse outcomes.^6^, ^7^, ^8^, ^9^ Our previous minute-level ROSC trajectory paper made a related point from the parent dataset: early post-ROSC haemodynamics are dynamic, and brief hypotension in the first minutes after ROSC may matter.^7^ The present analysis extends that work by re-anchoring time on infusion initiation and showing that infusion start usually occurred during a downward trajectory rather than during a flat or already recovering phase.

We have deliberately avoided drawing strong parallels with ICU MAP-target trials. Those trials address different treatment strategies in a later, more monitored setting, whereas this study describes the immediate prehospital period around a clinician-initiated infusion start. The relevant contribution here is therefore not evidence for a higher MAP target, nor evidence that adrenaline infusion improves outcomes, but high-resolution information about the arterial-pressure pattern that future prehospital vasopressor trials may need to anticipate.

The next question is how consistent that average pattern was across patients. Only 46.8% met the MAP-rise definition, and change distributions were broad. Although 74.5% of patients had an improved MAP trajectory, fewer than half had a MAP rise of at least 5 mmHg by approximately 10 min. This means that, in some patients, MAP changed direction but did not produce a larger early pressure increase. Patients with a MAP rise started from lower pressures and then rose more steeply after infusion start. This heterogeneity is biologically plausible in post-resuscitation shock, which reflects varying contributions from myocardial dysfunction, vasoplegia, relative hypovolaemia, and evolving shock severity, with additional variation introduced by treatment selection and co-interventions.^1^, ^2^, ^6^ These subgroup summaries are descriptive rather than causal. Even so, this variability matters for future trials because it affects patient selection, expected response size, and outcome timing. The exploratory subgroup findings support this interpretation, but they are descriptive only. Patients in higher documented infusion-rate categories had larger pressure rises, but this should be interpreted as dose context, not evidence that higher infusion rates caused the rise, because rates were chosen by clinicians and were available as patient-level summaries rather than verified minute-by-minute pump titration data.

These limits are why this paper was deliberately framed as an around-start pharmacodynamic analysis rather than as an infusion-versus-no-infusion comparison. Infusion initiation followed evolving blood pressure, recent trajectory, prior bolus use, fluid administration, airway interventions, and clinician judgement. Under those conditions, broader between-group comparisons would be highly vulnerable to confounding by indication, time-dependent bias, immortal-time related problems, and weak support for a meaningful untreated comparator in some severity strata.^10^, ^14^ A causal question about infusion versus no infusion, and secondarily earlier versus later infusion, would require a design that defines eligibility, time zero, comparator strategies, and risk sets explicitly, and that handles blood pressure as both a determinant of treatment and a variable affected by previous treatment. It would also require richer measurement of acute post-ROSC shock severity than is routinely available in registry and electronic ambulance records, including markers of acidosis, vasoplegia, myocardial dysfunction, hypovolaemia, and evolving co-interventions.^14^ The current analysis was not designed for that purpose; it was designed to describe physiology immediately surrounding documented infusion start among patients who received the infusion.

This study’s practical contribution is physiological and trial-methodological. It provides a minute-level description of the prehospital post-ROSC phase, showing that adrenaline infusion was usually started during a falling blood pressure trajectory that then reversed over the next 20 min. For clinicians and trialists, the key point is that the typical signal was not an immediate jump at minute zero, but a turn in trajectory over the following several minutes. Infusion often began when pressures were still near or above conventional hypotension thresholds. This is clinically plausible because paramedics may respond to a falling blood pressure trend, recent bolus requirement, or a protocol goal such as maintaining SBP around 100 mmHg, rather than waiting for a single threshold crossing. Together, these findings add higher-resolution prehospital data to a literature that has largely relied on single handover values or later ICU measurements.^3^, ^7^, ^8^, ^9^ This trajectory-based description also helps define what clinicians might reasonably expect to see in the minutes after infusion initiation. Whether earlier initiation, different targets, or different vasopressor strategies improve patient-centred outcomes remains uncertain.

### Limitations

This study has important limitations. It does not identify the causal effect of adrenaline infusion. Infusion initiation was closely linked to evolving illness severity, recent trajectory, and clinician judgement, leaving significant scope for confounding by indication despite the within-patient design. Infusion-start timestamps were also likely imprecise, and the positive fake-start analyses show that the observed reversal was not tightly confined to a single recorded minute. Regression to the mean and broader around-start recovery remain plausible contributors to the signal. Blood pressure was measured mainly with intermittent non-invasive monitoring rather than continuous invasive tracing, which limited temporal precision.

Non-invasive cuff readings may be less reliable during hypotension, poor perfusion, movement, or transport, and we lacked calibration data or paired invasive pressures. We also lacked detailed pump mechanics, minute-by-minute titration, post-start fluid dosing, cardiac output, myocardial work, and in-hospital haemodynamic data. Therefore, the study cannot determine whether the pressure rise reflected improved flow, vasoconstriction, increased cardiac work, concurrent care, or spontaneous recovery. Denominators varied across analyses because usable data requirements differed, and the cohort was restricted to patients with linked monitor data, documented infusion start within 60 min of ROSC, and pulse present on hospital arrival. Taken together, these limitations mean the study supports a consistent around-start reversal in blood pressure trajectory, but not a clean causal treatment effect of adrenaline infusion.

## Conclusion

In this selected cohort of prehospital ROSC patients started on adrenaline infusion, MAP and SBP trajectories commonly changed from decline to subsequent rise around the documented infusion-start period. The average arterial-pressure signal was consistent, but individual responses were highly variable and dose titration, flow, and cardiac work could not be directly assessed. These findings improve description of dynamic prehospital post-ROSC adrenaline arterial-pressure responsiveness, but they do not establish that adrenaline infusion improves survival or neurological outcome. Further protocolised or explicitly causal studies are needed to test whether vasopressor timing, target, dose, or agent improves patient-centred outcomes.

## Data sharing statement

This study is a registry-based analysis of linked Ambulance Victoria and ZOLL® monitor data. All aggregated data relevant to the study are included in the article or uploaded as supplementary information. De-identified raw data are not publicly available due to privacy and governance restrictions but may be available from the corresponding author upon reasonable request and subject to approval by Ambulance Victoria and relevant ethics committees.

## CRediT authorship contribution statement

**Pieter Francsois Fouche:** Conceptualization, Formal analysis, Methodology, Writing – original draft, Writing – review & editing. **Emily Nehme:** Conceptualization, Data curation, Writing – review & editing. **Sam Burton:** Data curation, Writing – review & editing. **Benjamin Meadley:** . **David Anderson:** Resources, Writing – review & editing. **Belinda Flanagan:** Resources, Writing – review & editing. **Dion Stub:** Resources, Writing – review & editing. **Ziad Nehme:** Conceptualization, Investigation, Methodology, Supervision, Writing – original draft, Writing – review & editing.

## Funding

This research did not receive any specific grant from funding agencies in the public, commercial, or not-for-profit sectors.

## Declaration of competing interest

Professor Ziad Nehme is an Editorial Board Member of Resuscitation Plus. Given this role, Professor Nehme had no involvement in the peer review or editorial handling of this article and had no access to information regarding its peer review. Full responsibility for the editorial process for this article was delegated to another journal editor. The other authors declare that they have no known competing financial interests or personal relationships that could have appeared to influence the work reported in this paper.

## Appendix A Supplementary material

Supplementary Tables Additional methods, tables, figures, sensitivity analyses and model diagnostics supporting the main article.
