# Supplementary material for: Haemodynamic trajectories around prehospital adrenaline infusion start after return of spontaneous circulation
Source: Resusc Plus. 2026 May 21;30:101367. doi: 10.1016/j.resplu.2026.101367 (PMC13240826; doi:10.1016/j.resplu.2026.101367)
Supplement: Supplementary Tables — Additional methods, tables, figures, sensitivity analyses and model diagnostics supporting the main article. [file mmc1.docx]

*Supplementary Table S1. Infusion delivery and concurrent treatments at or before infusion start*

| **Variable** | **Infusion-starter cohort (N = 1,920)** |
| --- | --- |
| **Infusion delivery** | |
| Time from ROSC to infusion start, min | 11 (6-20) |
| Total prehospital adrenaline bolus dose, mg | 2.30 (1.05-4.10) |
| Any prior adrenaline bolus by start | 1,303 (67.9) |
| Adrenaline bolus in prior 5 min | 1,076 (56.0) |
| Cumulative bolus dose by start, mg | 0.06 (0.00-0.40) |
| Patient-level median documented infusion rate, mcg/min | 20 (10-50) |
| Documented numeric infusion-rate entries, n | 1 (1-3) |
| **Concurrent treatments at or before infusion start** | |
| Any crystalloid before start | 969 (50.5) |
| Crystalloid in prior 5 min | 148 (7.7) |
| Intubated before start | 1,519 (79.1) |
| Rocuronium, midazolam, ketamine, fentanyl before start | 527 (27.4) |
| Rocuronium, midazolam, ketamine, or fentanyl bolus in prior 5 min | 95 (4.9) |
| Amiodarone administered during encounter | 449 (23.4) |

Values are median (IQR) or n (%). Percentages are column percentages. The patient-level median documented infusion rate was calculated as the median of all numeric medication-record infusion-rate entries recorded for each patient. Documented numeric infusion-rate entries counts the number of numeric rate entries per patient. “Recent” was defined as within 5 minutes before the documented infusion-start minute.

*Supplementary Table S2. Minute-binned and slope-change summaries around infusion start*

| **Metric** | **N** | **Mean (SD)** | **Median (IQR)** |
| --- | --- | --- | --- |
| **Minute-binned change relative to pre-window mean pressure** | | | |
| ∆ MAP at +5 min, mmHg | 1,328 | -4.29 (27.37) | -5.32 (-21.11-11.51) |
| ∆ MAP at +10 min, mmHg | 1,341 | 4.03 (30.16) | 3.00 (-15.00-22.58) |
| ∆ MAP at +15 min, mmHg | 1,310 | 8.01 (30.37) | 7.83 (-11.33-28.10) |
| ∆ MAP at +20 min, mmHg | 1,204 | 10.39 (31.65) | 9.64 (-9.60-30.85) |
| ∆ SBP at +5 min, mmHg | 1,345 | -6.30 (38.53) | -7.93 (-30.00-17.00) |
| ∆ SBP at +10 min, mmHg | 1,350 | 5.73 (42.16) | 4.00 (-20.93-32.00) |
| ∆ SBP at +15 min, mmHg | 1,325 | 11.67 (42.86) | 11.54 (-15.50-40.00) |
| ∆ SBP at +20 min, mmHg | 1,210 | 15.69 (43.53) | 14.13 (-13.10-44.78) |
| **Slope-change summaries** | | | |
| MAP slope change, mmHg/min | 1,367 | 2.93 (6.02) | 2.39 (-0.02-5.81) |
| SBP slope change, mmHg/min | 1,373 | 4.33 (8.24) | 3.70 (0.37-8.40) |

∆ values are referenced to the patient-specific pre-window mean pressure. Minute-binned summaries were calculated from small bands around the target minute.

*Supplementary Table S3. Characteristics by MAP-rise definition at approximately +10 minutes*

| **Variable** | **MAP rise ≥5 mmHg (N = 627)** | **MAP rise <5 mmHg (N = 714)** |
| --- | --- | --- |
| **Patient and arrest characteristics** | | |
| Age, years | 63 (50-75) | 67 (57-77) |
| Male sex | 425 (67.8) | 483 (67.6) |
| Downtime, min | 28 (22-37) | 29 (22-38) |
| Public location | 110 (17.5) | 145 (20.3) |
| **Witness status** | | |
| EMS witnessed | 70 (11.2) | 105 (14.7) |
| Not witnessed | 180 (28.8) | 162 (22.8) |
| Public witness | 376 (60.1) | 445 (62.5) |
| **Initial rhythm** | | |
| VF/VT | 253 (40.4) | 302 (42.5) |
| PEA | 163 (26.0) | 207 (29.1) |
| Asystole | 182 (29.0) | 184 (25.9) |
| Non-shockable/other | 29 (4.6) | 18 (2.5) |
| Presumed cardiac cause | 442 (70.5) | 551 (77.2) |
| **Treatment context at infusion start** | | |
| Time from ROSC to infusion start, min | 12 (8-22) | 11 (6-22) |
| Any prior adrenaline bolus by start | 491 (78.3) | 431 (60.4) |
| Cumulative bolus dose by start, mg | 0.10 (0.02-0.50) | 0.05 (0.00-0.35) |
| Any crystalloid before start | 328 (52.3) | 371 (52.0) |
| Intubated before start | 489 (78.0) | 560 (78.4) |
| Rocuronium, midazolam, ketamine, fentanyl before start | 163 (26.0) | 238 (33.3) |
| **Pre-infusion haemodynamics** | | |
| Pre-window mean MAP, mmHg | 73.5 (60.0-85.3) | 93.6 (80.6-106.1) |
| Pre-window mean SBP, mmHg | 98.2 (79.5-115.0) | 125.4 (106.9-144.5) |
| Pre-infusion MAP slope, mmHg/min | -0.79 (-3.39-0.39) | -1.60 (-4.83-0.00) |
| Pre-infusion SBP slope, mmHg/min | -1.27 (-4.85-0.34) | -2.56 (-7.00-0.00) |

MAP-rise category was defined among 1,341 patients with ∆ MAP at approximately +10 minutes available. Values are median (IQR) or n (%); percentages are column percentages. Available denominators differed from the header N for downtime (627/713), witness status (626/712), initial rhythm (627/711), pre-infusion MAP slope (587/657), and pre-infusion SBP slope (587/657). This table is descriptive only because the grouping variable is defined after infusion initiation.

*Supplementary Table S4. Lagged concurrent-treatment terms from the sensitivity models*

| **Covariate** | **Coefficient (95% CI)** | **p-value** |
| --- | --- | --- |
| **MAP lagged co-intervention terms** | | |
| Recent adrenaline bolus, prior 5 min (1-minute lag) | -5.54 (-6.79 to -4.28) | <0.001 |
| Recent crystalloid proxy, prior 5 min (1-minute lag) | -2.27 (-4.43 to -0.11) | 0.040 |
| Recent intubation, prior 5 min (1-minute lag) | 0.73 (-1.30 to 2.76) | 0.479 |
| Recent sedative/paralytic, prior 5 min (1-minute lag) | 2.80 (1.08 to 4.53) | 0.001 |
| **SBP lagged co-intervention terms** | | |
| Recent adrenaline bolus, prior 5 min (1-minute lag) | -7.68 (-9.43 to -5.94) | <0.001 |
| Recent crystalloid proxy, prior 5 min (1-minute lag) | -2.68 (-5.70 to 0.35) | 0.083 |
| Recent intubation, prior 5 min (1-minute lag) | 1.00 (-1.91 to 3.90) | 0.501 |
| Recent sedative/paralytic exposure, prior 5 min (1-minute lag) | 3.49 (1.07 to 5.91) | 0.005 |

These coefficients come from the lagged sensitivity models and are reported to show how recent concurrent care related to the observed around-start blood pressure trajectories. They should not be interpreted as causal treatment effects.

*Supplementary Table S5. Placebo-breakpoint, window-robustness, and fake-start checks*

| **Check** | **Estimate (95% CI)** | **p-value** |
| --- | --- | --- |
| **Pre-window slope checks** | | |
| **MAP** | | |
| Early pre-window slope (-10 to -6 min) | -1.48 (-2.18 to -0.78) | <0.001 |
| Late pre-window slope (-5 to -1 min) | -1.90 (-2.40 to -1.40) | <0.001 |
| **SBP** | | |
| Early pre-window slope (-10 to -6 min) | -1.73 (-2.69 to -0.77) | <0.001 |
| Late pre-window slope (-5 to -1 min) | -2.70 (-3.39 to -2.01) | <0.001 |
| **Placebo-breakpoint checks** | | |
| **MAP** | | |
| Placebo pseudo slope change | -0.38 (-1.15 to 0.39) | 0.328 |
| Placebo pseudo level change | -0.45 (-2.12 to 1.22) | 0.596 |
| **SBP** | | |
| Placebo pseudo slope change | -0.66 (-1.71 to 0.39) | 0.216 |
| Placebo pseudo level change | -0.76 (-2.95 to 1.43) | 0.495 |
| **Window-robustness checks, slope change term** | | |
| **MAP** | | |
| -5 to +15 window | 3.37 (2.88 to 3.87) | <0.001 |
| -10 to +15 window | 2.97 (2.65 to 3.28) | <0.001 |
| -10 to +30 window | 2.49 (2.20 to 2.78) | <0.001 |
| **SBP** | | |
| -5 to +15 window | 4.84 (4.15 to 5.54) | <0.001 |
| -10 to +15 window | 4.27 (3.82 to 4.71) | <0.001 |
| -10 to +30 window | 3.51 (3.10 to 3.91) | <0.001 |
| **Fake-start checks, slope change term** | | |
| **MAP** | | |
| Breakpoint shifted 3 min earlier | 2.53 (2.19 to 2.87) | <0.001 |
| Breakpoint shifted 5 min earlier | 2.00 (1.61 to 2.38) | <0.001 |
| **SBP** | | |
| Breakpoint shifted 3 min earlier | 3.52 (3.06 to 3.99) | <0.001 |
| Breakpoint shifted 5 min earlier | 2.85 (2.32 to 3.39) | <0.001 |

The placebo model inserted a pseudo breakpoint at -5 minutes within the pre-infusion window. Window-robustness rows report the slope-change term from alternative around-start windows. Fake-start rows report the slope-change term after shifting the breakpoint 3 or 5 minutes earlier than the documented infusion start.

*Supplementary Table S6. Exploratory subgroup summaries of early MAP response around infusion start*

| **Subgroup** | **N** | **MAP rise ≥5 mmHg, %** | **ΔMAP +10 min, mean (SD), mmHg** | **Positive MAP slope change, %** |
| --- | --- | --- | --- | --- |
| **Pre-infusion MAP category** | | | | |
| MAP <65 mmHg | 266 | 85.6 | 29.1 (24.3) | 71.0 |
| MAP 65-69 mmHg | 97 | 56.8 | 11.2 (22.5) | 75.0 |
| MAP 70-84 mmHg | 407 | 56.6 | 10.7 (23.2) | 73.1 |
| MAP ≥85 mmHg | 735 | 25.6 | -9.9 (29.0) | 76.3 |
| **Pre-infusion MAP slope category** | | | | |
| Falling >1 mmHg/min | 715 | 44.0 | 2.9 (28.9) | 98.6 |
| Stable -1 to +1 mmHg/min | 433 | 46.9 | 3.3 (30.6) | 69.4 |
| Rising >1 mmHg/min | 243 | 56.6 | 11.6 (30.1) | 12.1 |
| **ROSC-to-infusion timing tertile** | | | | |
| Early tertile | 674 | 39.1 | -2.7 (28.0) | 73.1 |
| Middle tertile | 634 | 52.4 | 8.4 (32.7) | 79.4 |
| Late tertile | 612 | 46.8 | 4.7 (28.1) | 70.4 |
| **Cumulative post-ROSC adrenaline bolus dose quartile** | | | | |
| Bolus dose Q1 | 617 | 32.5 | -5.3 (26.5) | 77.6 |
| Bolus dose Q2 | 361 | 44.7 | 3.4 (29.5) | 77.9 |
| Bolus dose Q3 | 464 | 65.7 | 15.6 (28.2) | 73.7 |
| Bolus dose Q4 | 478 | 48.2 | 5.3 (32.8) | 68.4 |
| **Documented median infusion-rate category** | | | | |
| ≤10 micrograms/min | 708 | 37.4 | -0.9 (27.8) | 73.8 |
| 11-20 micrograms/min | 342 | 47.0 | 4.6 (28.5) | 75.5 |
| >20 micrograms/min | 870 | 56.2 | 8.9 (32.3) | 74.7 |
| **Prior post-ROSC adrenaline bolus** | | | | |
| No | 617 | 32.5 | -5.3 (26.5) | 77.6 |
| Yes | 1,303 | 53.3 | 8.3 (30.8) | 73.0 |
| **Recent adrenaline bolus, prior 5 min** | | | | |
| No | 844 | 35.0 | -3.6 (27.8) | 72.8 |
| Yes | 1,076 | 56.1 | 10.1 (30.6) | 75.8 |
| **Recent crystalloid, prior 5 min** | | | | |
| No | 1,772 | 46.2 | 3.6 (30.2) | 74.0 |
| Yes | 148 | 53.2 | 8.3 (29.8) | 79.6 |
| **Intubation before infusion start** | | | | |
| No | 401 | 47.3 | 5.9 (30.7) | 72.1 |
| Yes | 1,519 | 46.6 | 3.5 (30.0) | 75.2 |
| **Recent sedation or paralysis, prior 5 min** | | | | |
| No | 1,825 | 46.5 | 4.0 (30.0) | 74.4 |
| Yes | 95 | 51.4 | 4.2 (32.5) | 75.0 |

**Note.** Values are descriptive summaries by clinical context strata. N is the subgroup size. Derived blood-pressure summaries had varying denominators according to available pre- and post-infusion blood-pressure data. ΔMAP is the change from the patient-specific pre-window mean MAP to approximately +10 minutes after infusion start. Rate categories use patient-level median documented infusion-rate summaries and should not be interpreted as verified minute-by-minute pump titration or causal dose-response estimates.

*Supplementary Table S7. Implausible blood-pressure value counts in the linked ZOLL extract*

| **Variable** | **Rule** | **Records** | **Percentage of included ZOLL records** |
| --- | --- | --- | --- |
| All included ZOLL records | Records before invalid-value count | 553,177 | 100.000 |
| SBP | Non-positive SBP | 0 | 0.000 |
| DBP | Non-positive DBP | 10 | 0.002 |
| MAP | Non-positive MAP | 0 | 0.000 |

**Note.** Counts were calculated among ZOLL blood-pressure records linked to the infusion-starter cohort. Percentages use all included ZOLL blood-pressure records as the denominator. These counts describe non-positive values only and do not assess cuff calibration, cuff size, measurement duration, or agreement with invasive arterial pressure.
